# Enteroendocrine Cell Formation Is an Early Event in Pancreatic Tumorigenesis

**DOI:** 10.3389/fphys.2022.865452

**Published:** 2022-04-27

**Authors:** Leah R. Caplan, Vera Vavinskaya, David G. Gelikman, Nidhi Jyotsana, Vincent Q. Trinh, Kenneth P. Olive, Marcus C. B. Tan, Kathleen E. DelGiorno

**Affiliations:** ^1^ Department of Cell and Developmental Biology, Vanderbilt University, Nashville, TN, United States; ^2^ Department of Pathology, University of California, San Diego, San Diego, CA, United States; ^3^ College of Medicine, University of Central Florida, Orlando, FL, United States; ^4^ Department of Surgery, Vanderbilt University Medical Center, Nashville, TN, United States; ^5^ Department of Medicine, Herbert Irving Comprehensive Cancer Center, Columbia University Irving Medical Center, New York, NY, United States; ^6^ Vanderbilt Digestive Disease Research Center, Vanderbilt University Medical Center, Nashville, TN, United States; ^7^ Vanderbilt Ingram Cancer Center, Nashville, TN, United States; ^8^ Epithelial Biology Center, Vanderbilt University School of Medicine, Nashville, TN, United States

**Keywords:** enteroendocrine cells, pancreas, pancreatic polypeptide (PP), serotonin, ghrelin, somatostatin

## Abstract

Pancreatic ductal adenocarcinoma (PDAC) is a devastating disease with a 5-year survival rate of only 11%, due, in part, to late diagnosis, making the need to understand early events in tumorigenesis critical. Acinar-to-ductal metaplasia (ADM), when not resolved, is a PDAC precursor. Recently, we showed that ADM is constituted by a heterogenous population of cells, including hormone-producing enteroendocrine cells (EECs: gamma, delta, epsilon, and enterochromaffin cells). In this study, we employed histopathological techniques to identify and quantify the abundance of EEC subtypes throughout pancreatic tumorigenesis in mouse models and human disease. We found that EECs are most abundant in ADM and significantly decrease with lesion progression. Co-immunofluorescence identifies distinct lineages and bihormonal populations. Evaluation of EEC abundance in mice lacking *Pou2f3* demonstrates that the tuft cell master regulator transcription factor is not required for EEC formation. We compared these data to human neoplasia and PDAC and observed similar trends. Lastly, we confirm that EECs are a normal cellular compartment within the murine and human pancreatic ductal trees. Altogether, these data identify EECs as a cellular compartment of the normal pancreas, which expands early in tumorigenesis and is largely lost with disease progression.

## Introduction

Pancreatic ductal adenocarcinoma (PDAC) is a devastating disease and is predicted to become the second leading cause of cancer-related deaths by the year 2030 (Rahib et al., 2014). While the 5-year survival rate recently increased to 11%, progress is slow. This is due, in part, to late diagnosis and a lack of knowledge of early events in tumorigenesis. Acinar-to-ductal metaplasia (ADM) is a reparative program in which pancreatic acinar cells transdifferentiate into ductal cells in response to injury or oncogenic mutation (Giroux and Rustgi, 2017). Recently, we combined acinar cell lineage tracing in murine models and single cell RNA sequencing (scRNA-seq) and revealed that ADM does not result in the formation of homogeneous ductal cells but seeds a heterogeneous population that includes tuft cells and enteroendocrine cell (EEC) subtypes, identified by hormone expression (DelGiorno et al., 2020a; Ma et al., 2021). These ADM-derived EEC subtypes include gamma cells (pancreatic polypeptide, PP), delta cells (somatostatin, SST), epsilon cells (ghrelin, GHRL), and enterochromaffin cells (serotonin, 5-HT) (Ma et al., 2021). While these hormones are present in murine islet development and in normal human islets (to varying degrees), we define ADM populations as EECs due to incorporation into ducts and their similarity to analogous populations throughout the gut (Wierup et al., 2002; Ohta et al., 2011; Ma et al., 2021).

Under conditions of unresolved injury, ADM can serve as a precursor for PDAC. Progression is a response to oncogenic mutation(s), such as in *KRAS*, the most common mutation in human pancreatic cancer, which is sufficient to drive the formation of precancerous lesions like pancreatic intraepithelial neoplasia (PanIN) (Hingorani et al., 2003). Previous studies have documented endocrine-like cell formation in murine and human PanIN and PDAC, however these studies did not address the formation, abundance, and dynamics of EEC subtypes throughout pancreatic tumorigenesis (Chen et al., 1988; Farrell et al., 2017; Sinha et al., 2017). Here, we identified and quantified the abundance of each EEC subtype present in multiple autochthonous models of pancreatic tumorigenesis and compared these findings to EEC abundance in patient samples encompassing normal pancreas, ADM, PanIN, and PDAC. We found that EEC subtype abundance in mouse models is highest in ADM and significantly decreases with disease progression. This trend holds true in human disease, with divergence between mouse and human observed primarily in invasive adenocarcinoma. Further, we evaluated EEC subtype-specific hormone expression and found it to be largely subtype-restricted, apart from several bihormonal populations which have not previously been described in pancreatic tumorigenesis. We demonstrate that tuft cell master regulator transcription factor, POU2F3, is not required for EEC formation (Yamaguchi et al., 2014; Gerbe et al., 2016). Recently, we found that tuft cell formation inhibits pancreatic tumorigenesis by modulating the microenvironment with anti-inflammatory prostaglandins (DelGiorno et al., 2020b). These data demonstrate that ADM-derived populations can have a significant effect on disease progression, suggesting that EEC subtype formation and abundance could have a significant impact on tumor formation and severity. Therefore, studying disease-associated EEC subtypes may identify pathways to target or co-opt for patient benefit.

## Materials and Methods

### Mice

Mice were housed in accordance with NIH guidelines in AAALAC-accredited facilities at the Salk Institute for Biological Studies or Columbia University. The IACUC committees at the Salk Institute or Columbia University approved all animal studies. *LSL-Kras* ^*G12D/+*^ *; Ptf1a* ^*Cre/+*^ (*KC*), *LSL-Kras* ^*G12D/+*^ *;Pou2f3* ^*fl/fl*^ *; Ptf1a* ^*Cre/+*^ (*KPouC*), and *LSL-Kras* ^*G12D/+*^ *;Trp53* ^*R17H*^ *;Pdx1Cre* (*KPC*) mice have been described previously (Hingorani et al., 2003; Hingorani et al., 2005; DelGiorno et al., 2020b).

### Human Samples

Distribution and use of all human samples was approved by the Institutional Review Boards at Vanderbilt University and Vanderbilt University Medical Center.

### Histological Staining

Tissues were fixed overnight in zinc-containing, neutral-buffered formalin (Fisher Scientific), embedded in paraffin, cut in 5 μm sections, mounted, and stained. Sections were deparaffinized in xylene, rehydrated in a graded series of ethanols, and then washed in PBST and PBS. Endogenous peroxidase activity was blocked with a 1:50 solution of 30% H_2_O_2_:PBS followed by microwave antigen retrieval in 100 mM sodium citrate, pH 6.0. Sections were blocked with 1% bovine serum albumin (BSA) and 5% normal goat or rabbit serum in 10 mM Tris (pH 7.4), 100 mM MgCl_2_, and 0.5% Tween-20 for 1 h at room temperature. Primary antibodies were diluted in blocking solution and incubated overnight. Information on primary antibodies is provided in Supplementary Table S1. Slides were then washed, incubated in streptavidin-conjugated secondaries (for rabbit or mouse antibodies, Abcam, for rat or goat antibodies, Vector) and developed with DAB substrate (Vector). Immunofluorescence on paraffin-embedded tissues followed the immunohistochemistry protocol until the blocking step. Instead, tissues were blocked with 5% normal donkey serum and 1% BSA in 10 mM PBS for 1 h at room temperature. Tissue sections were stained with primary antibodies in 10 mM PBS supplemented with 1% BSA and 0.1% Triton X-100 overnight (Supplementary Table S1). Sections were then washed 3 × 15 min in PBS with 1% Triton X-100, incubated in Alexa Fluor secondary antibodies, washed again for 3 × 5 min, rinsed with distilled water, and mounted with Prolong Gold containing Dapi (Invitrogen). All slides were scanned and imaged on an Olympus VS-200 Virtual Slide Scanning microscope.

### Murine Co-Immunofluorescence Quantification

Slides from normal C57 BL/6 and CD1 mice were stained with antibody panels to quantify the presence of hormone+ cells in normal murine ducts. Due to their rare occurrence, only the number of intra- and interlobular ducts harboring a hormone+ cell were quantified, as opposed calculating the percentage of hormone+ cell(s) relative to the total number of cells per duct. Only ductal structures with clear lumens were counted. Intercalated ducts were excluded due to their variability in 2D appearance. The common bile duct was excluded due to its different morphology, function, and similarity to the small intestine.

Slides from 6-month-old *KC* mice were stained with antibody panels to determine if single EECs express multiple hormones or if hormones are EEC subtype restricted. Stained slides were imaged using identical imaging parameters to compare results between samples. To control for autofluorescence or channel bleed through, a minimum intensity threshold was determined for each antibody for each staining combination. Positive cells were identified in luminal and PanIN structures, excluding islets or islets closely adjacent to ductal lesions. For hormone co-expression analysis, 100 positive cells were identified for each hormone per mouse (*n* = 3) independent of the co-stained hormone. Of note, only 236 gastrin+ cells were identified (per mouse = 100, 100, 36 gastrin+ cells). Next, the cells positive for each hormone were individually assessed for co-positivity based on the previously set minimum thresholding. The number of co-positive cells were combined, and the percentage of co-positivity was calculated.

### Pathological Scoring of Murine Samples

Stained slides from *KC*, *KPouC*, or *KPC* mice were scanned on an Olympus VS-200 Virtual Slide Scanning microscope and approximately 10, 10× images were captured per mouse per stain (synaptophysin, somatostatin, pancreatic polypeptide, serotonin, and ghrelin). Lesions with or without staining (1–6 per image depending on abundance) were randomly chosen and were then graded by a pathologist (VV). The number of positively stained cells and nuclei in each lesion was then counted and the percentage of positive cells in that lesion determined.

A second pathologist subspecialized in liver and pancreas diseases (VQT) blindly scored 114 previously scored regions of interest. Concordance was noted for 72% of ROIs, and discordances never surpassed one grade. Interobserver agreement was tested in SPSS version 26 by transforming ADM, PanIN-1A, PanIN-1B, PanIN-2, PanIN-3, and invasive cancer into 1-2-3-4-5-6. Cohen’s κ coefficient was 0.633 (ASD = 0.053), interpreted as substantial interobserver agreement. This suggests that the first pathologist’s scoring is reliable and constant, as prior studies have noted κ coefficients ranging from 0.13 to 0.43 in prior studies of PanIN interobserver variability (Hruban et al., 2001; Longnecker et al., 2005).

### Pathological Scoring of Patient Samples

Multiple samples from 11 patients with PDAC (*n* = 21 total slides) were stained for EEC hormones and analyzed individually. Hormone+ cells (GHRL, SST, 5-HT, and PP) were identified in lesions with ductal morphology; acinar and islet-associated hormone+ cells were excluded from analysis. Lesions harboring hormone+ cell(s) were then graded by a pathologist (VQT) using both the stained slide and an H&E stain from the same block. The lesion and the surrounding area were graded and recorded. Morphologically normal pancreatic ducts were graded and recorded as either normal or reactive. Lesions were classified as ADM, PanIN1a, PanIN1b, PanIN2, PanIN3, or invasive adenocarcinoma. The total number of lesions harboring at least one hormone+ cell was calculated (e.g. 268 SST+ lesions were identified in 21 slides). The percentage of each lesion grade relative to the total number of lesions was calculated (e.g. 76 of the 268 SST+ cells are found in ADM which is 28.4% of SST+ lesions).

### Statistical Analysis

Statistical analyses were performed in Prism (GraphPad). Statistical significance was calculated by either two-tailed unpaired t-tests assuming equal variance or one-way ANOVA. Data are expressed as mean ± standard deviation.

### Image Processing

Images were captured with an Olympus VS-200 Virtual Slide Scanning microscope and processed using ImageJ or Fiji. Figures were made using Adobe Photoshop and Illustrator.

## Results

### Enteroendocrine Cells Are a Cellular Compartment of the Normal Pancreas

**FIGURE 1:**
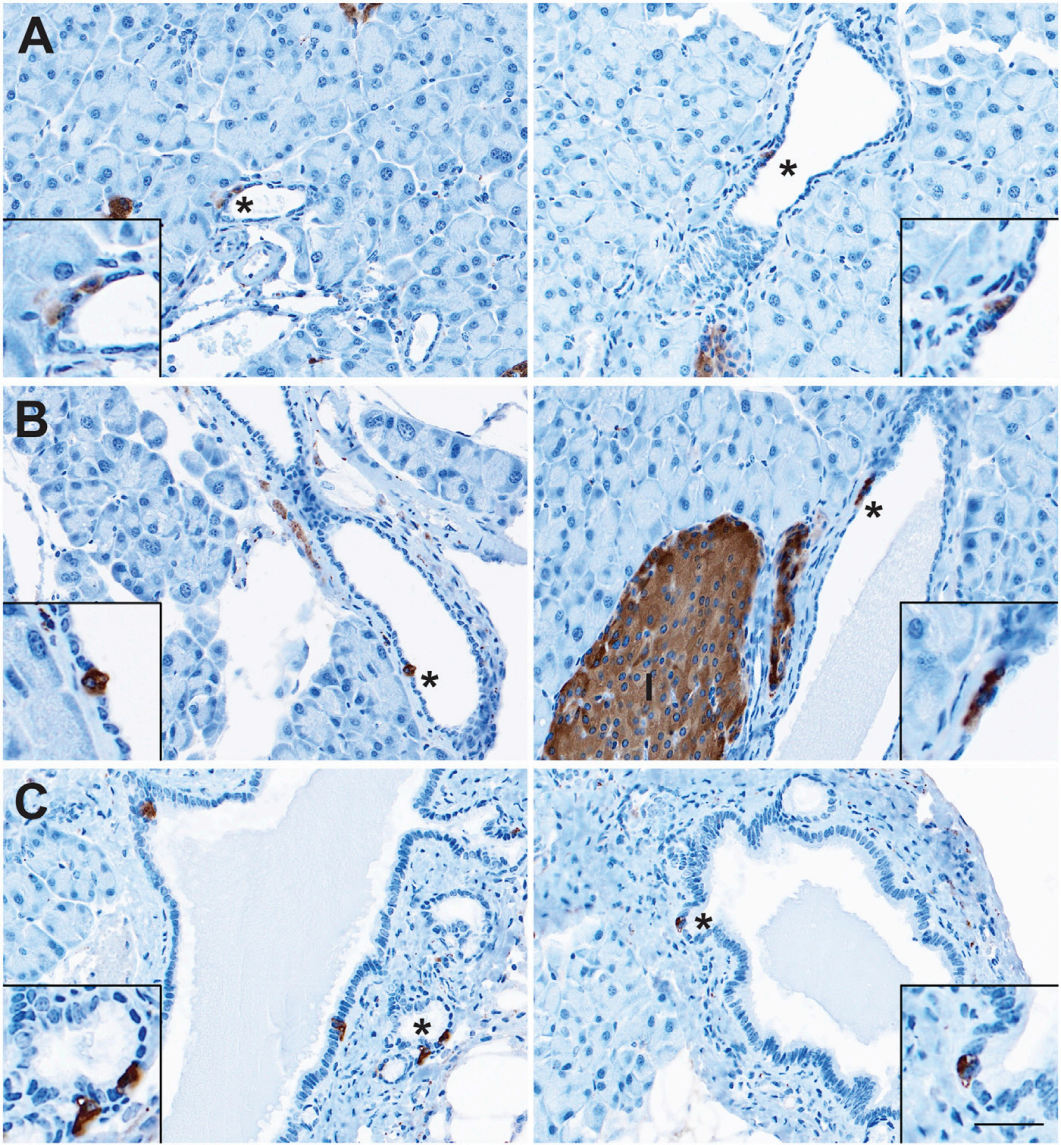
Enteroendocrine cells as a cellular compartment of the normal murine pancreas. Immunohistochemistry for synaptophysin (brown) in the **(A)** small and **(B)** large ducts of the normal pancreas, as well as in **(C)** the pancreatobiliary duct. I, islet. Scale bar, 50 μm.

The presence of sporadic cells expressing islet-associated hormones (insulin [INS], glucagon [GCG], SST, and PP) within the normal pancreatic and pancreatobiliary ducts of rats and humans has been reported on for decades (Chen et al., 1988; Park and Bendayan, 1992; Bouwens and Pipeleers, 1998; Li et al., 2016). However, some of these populations may represent ductal-like enteroendocrine cells (EECs) similar to those found throughout the gut rather than solitary islet cells. To confirm the presence of solitary islet cells or EECs in the murine pancreatic ductal tree, we conducted immunohistochemistry (IHC) for pan-endocrine marker, synaptophysin (SYP), on normal pancreata from CD1 (*n* = 5) and C57 BL/6 (*n* = 5) mice and examined expression throughout the ducts (Figure 1). We observed solitary SYP+ cells within the epithelial lining of small and large ducts, as well as in the pancreatobiliary duct, which forms from the convergence of the main pancreatic duct and the common bile duct and empties into the duodenum (Figure 1).

**FIGURE 2:**
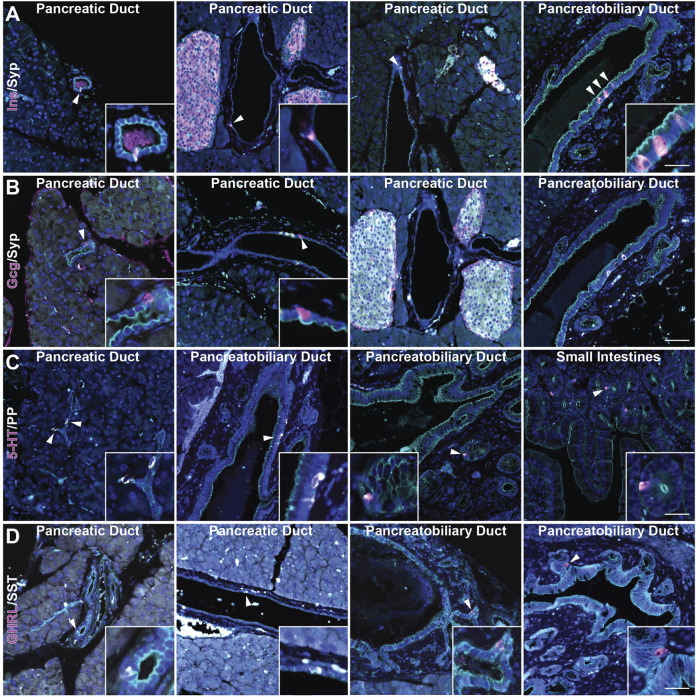
Characterization of enteroendocrine cell subtypes in the normal murine pancreas. Co-immunofluorescence for DAPI (blue), γ Actin (cyan) and **(A)** insulin (Ins, magenta) and synaptophysin (Syp, white), **(B)** glucagon (Gcg, magenta) and synaptophysin (Syp, white), **(C)** serotonin (5-HT, magenta) and PP (white), and **(D)** ghrelin (GHRL, magenta) and SST (white) in ducts of pancreas, including the pancreatobiliary duct, or small intestines. Scale bar, 50 μm.

Recently, we reported the formation of multiple EEC subtypes arising in injury-induced ADM (Ma et al., 2021). These EECs include archetypal islet cell types (gamma and delta cells expressing pancreatic polypeptide (PP) and somatostatin (SST), respectively), as well as EECs found in the epithelial lining of the stomach and intestines (delta cells, enterochromaffin cells expressing (5-HT), and epsilon cells expressing ghrelin (GHRL)) (Andersson-Rolf et al., 2000). While the presence of gamma, delta, and enterochromaffin cells in the normal rat pancreatic ductal epithelium has been described, epsilon cell formation has not and studies in the murine ductal tree are lacking (Park and Bendayan, 1992). To assay for these cell types in normal murine pancreatic ducts, we performed co-immunofluorescence (co-IF) for the previously listed archetypal islet cell types including insulin (INS), glucagon (GCG), SST, and PP, as well as serotonin (5-HT) and ghrelin (GHRL)+ EECs, and cell membrane marker γ-actin, to confirm the localization of these cells within the ducts (Figure 2). Consistent with previous reports, we observed INS+ and GCG+ cells in intra- and interlobular ducts (Figures 2A,B). INS+ cells were also observed in the pancreatobiliary duct, however GCG+ cells were not (Figures 2A,B). Additionally, INS+ cells, but not GCG+ cells, were co-positive for SYP (Figure 2B and Supplementary Figure S1). PP+ and SST+ cells were observed in intercalated, intra- and interlobular, and pancreatobiliary ducts consistent with previous reports (Figures 2C,D) (Park and Bendayan, 1992; Bertelli et al., 1994). Interestingly, 5-HT+ and GHRL+ cells were mainly observed in the pancreatobiliary duct and associated peribiliary glands, terminal structures that bud from the main ducts located in the head of the pancreas (Figures 2C,D). To assess the proportion of normal murine pancreatic ducts containing hormone-expressing cells, we calculated the percentage of intra- and interlobular ducts harboring at least one SST+, PP+, 5-HT+ or GHRL+ cell. SST+ cells were found in 2.34% of ducts (*n* = 683), and PP+ cells were found in 2.86% of normal ducts (*n* = 593) (Supplementary File S1). Alternatively, 5-HT+ and GHRL+ cells were not observed in any of the analyzed inter- and intralobular ducts (*n* = 593 and 683, respectively) (Supplementary File S1). These observations are consistent with previous studies of the pancreatic ductal tree in humans and other model systems, but also confirm the presence of EECs in the murine ductal tree and describe the presence of GHRL+ and 5-HT+ cells as largely restricted to the pancreatobiliary duct in mice.

### Synaptophysin Expression Is Most Abundant in Early Stages of Tumorigenesis

**FIGURE 3:**
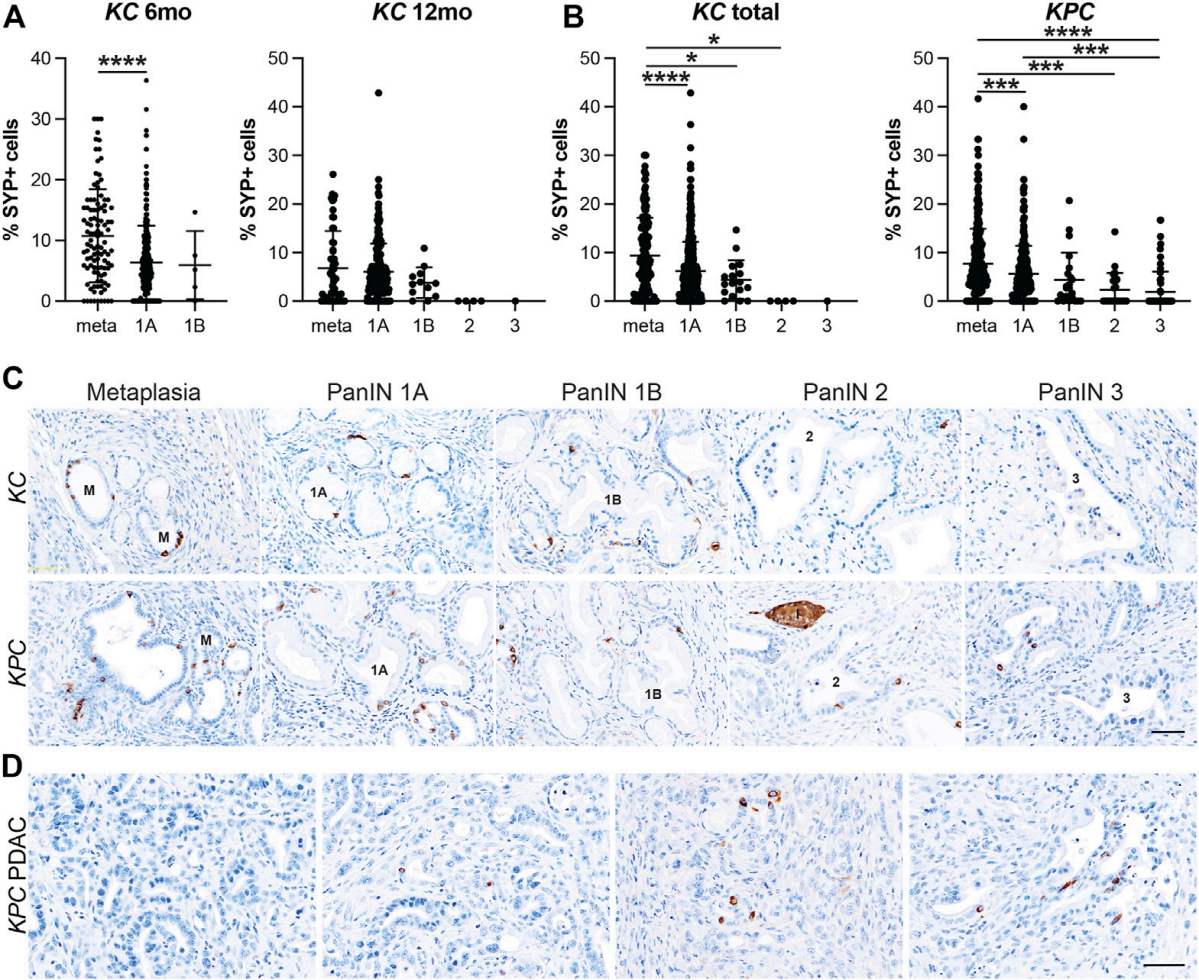
Enteroendocrine cell abundance decreases throughout pancreatic tumorigenesis. Quantification of EECs as the percentage of synaptophysin (SYP) positive cells per lesion in metaplastic ducts (meta) through increasing grades of PanIN (1A-3) in **(A)** 6- or 12-month old *KC* mice or **(B)** *KPC* mice in various stages of disease progression. *p < 0.05; ***p < 0.005; ****p < 0.001. **(C)** Representative SYP IHC of lesions of increasing grade from either *KC* or *KPC* mice. I, islet. **(D)** IHC for SYP+ or SYP-adenocarcinoma in *KPC* mice. Scale bar, 50 μm.

We have recently shown that EECs arise in the injured pancreas during ADM (Ma et al., 2021). To determine when EEC formation occurs in pancreatic tumorigenesis, we conducted IHC on pancreas tissue from 6 or 12-month old *Kras* ^*G12D*^ *;Ptf1a* ^*Cre/+*^ (*KC*) mice and *Kras* ^*G12D*^ *;Trp53* ^*R172H*^ *;Pdx1-Cre* (*KPC*) mice with or without PDAC for pan-EEC marker SYP. Multiple regions of interest were chosen and lesions spanning pancreatic tumorigenesis (metaplasia, PanIN1a, PanIN1b, PanIN2, PanIN3, and PDAC) were graded by a pathologist. The proportion of SYP-positive cells was then calculated as well as the total number of nuclei (hematoxylin) in each lesion to determine the relative abundance of EECs. In total, 690 and 689 lesions from *KC* and *KPC* mice, respectively, were analyzed. In 6-month-old *KC* pancreata (*n* = 7 mice), metaplastic ducts were found to contain a significantly higher proportion of SYP+ cells as compared to PanIN1a lesions (10.76%, *n* = 105 vs 6.35%, *n* = 302; *p* < 0.001) (Figures 3A,C and Supplementary File S1). As 6-month-old *KC* mice typically do not develop high-grade lesions, we next examined pancreata from mice aged 12 months (*n* = 5) where more PanIN1b and PanIN2 lesions were observed. Consistent with patterns identified in 6-month-old *KC* mice, we found SYP+ cells to be most abundant in metaplasia (6.82%, *n* = 58), decreasing with increasing PanIN grade (PanIN1a, 6.00%, *n* = 203; PanIN1b, 3.79%, *n* = 12; PanIN2, 0%, *n* = 4; PanIN3, 0%, *n* = 1) (Figures 3A,C and Supplementary File S1). SYP+ cells are more abundant in 6-month-old than in 12-month-old *KC* pancreata in lesions of equivalent grade (Supplementary Figure S2A). To examine more high-grade lesions and PDAC specimens, we next extended our analysis to pancreata from *KPC* mice (*n* = 19).

Similar to the *KC* dataset, the *KPC* dataset is also composed primarily of metaplasia and PanIN1a, but additionally harbors more high-grade lesions and PDAC, presenting a more complete distribution of lesion grades throughout pancreatic tumorigenesis. In total, pancreata from 19 *KPC* mice were analyzed including 10 mice with cancer. As shown in Figures 3B–C, SYP+ cells are most abundant in metaplasia (7.72%, *n* = 301) and decrease with increasing lesion grade (PanIN1a, 5.59%, *n* = 281; PanIN1b, 4.39%, *n* = 22; PanIN2, 2.32%, *n* = 24; PanIN3, 1.89%, *n* = 61). Of the 10 mice with cancer, only three pancreata had SYP+ cells associated with PDAC. Whether these cells are cancerous or are associated low grade lesions enveloped by the cancerous tissue is unclear (Figure 3D). Interestingly, we found that SYP+ cells are significantly more abundant in metaplasia in *KC* mice than in *KPC* mice (9.36%, *n* = 163 vs 7.72%, *n* = 301; *p* < 0.05), although SYP+ cells are more abundant in *KPC* pancreata later in disease progression (Supplementary Figure S2B). Altogether, analysis of SYP expression in the *KC* and *KPC* datasets demonstrates that EECs are significantly more prevalent in low-grade lesions consistent with EEC formation as an early event in tumorigenesis.

### EEC Subtype Dynamics Throughout Pancreatic Tumorigenesis

**FIGURE 4:**
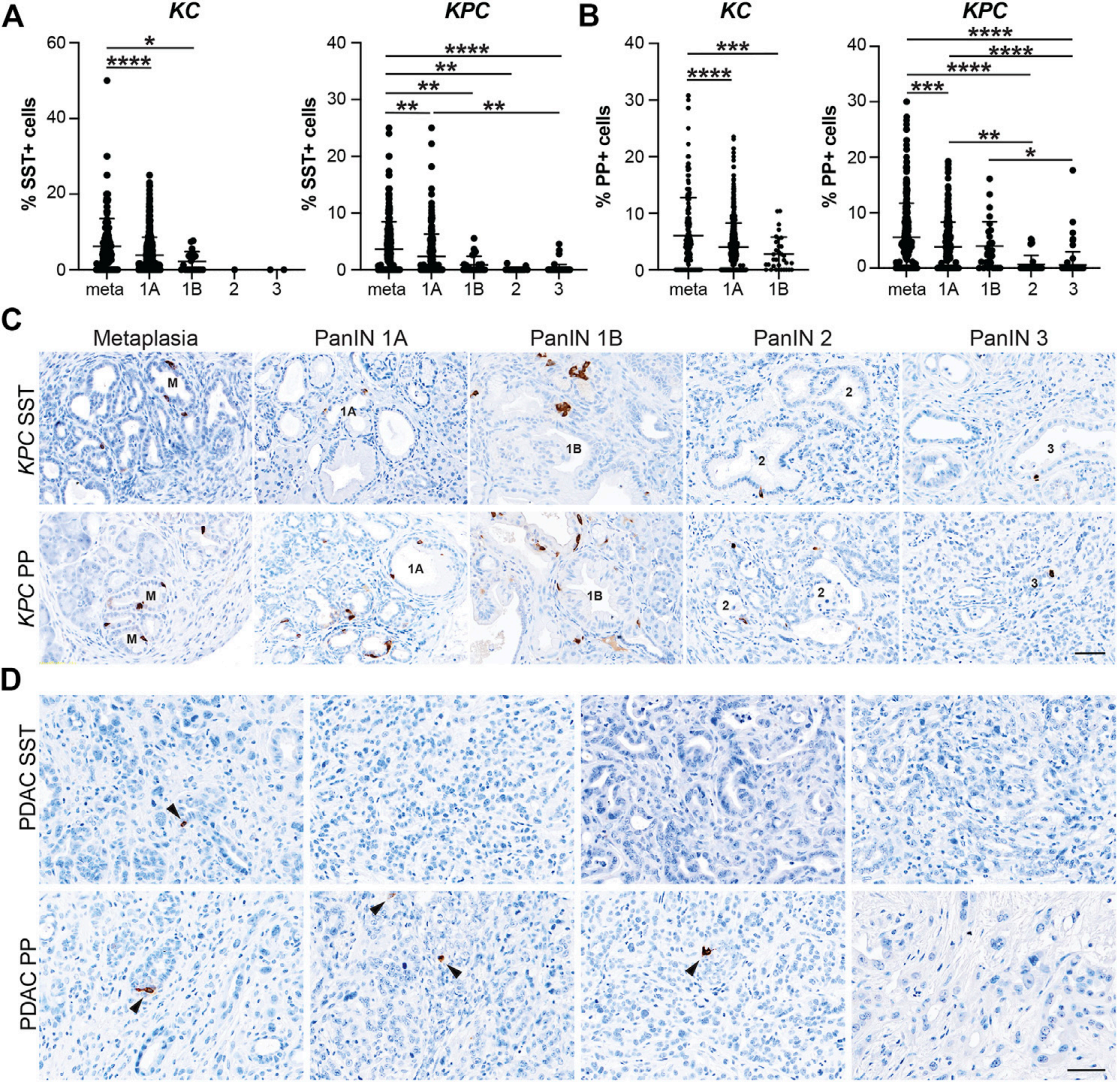
Delta and gamma cell abundance throughout pancreatic tumorigenesis. Quantification of **(A)** delta cells (SST+) or **(B)** gamma cells (PP+) in lesions from *KC* or *KPC* mice. *p < 0.05; **p < 0.01, ***p < 0.005; ****p < 0.001. **(C)** Representative SST or PP IHC of lesions of increasing grade in *KPC* mice. **(D)** IHC for SST or PP in adenocarcinoma in *KPC* mice. Arrows, positive cells. Scale bar, 50 μm.

After confirming that injury-induced EEC subtypes are present in neoplasia, we sought to determine the relative abundance of these subtypes and their temporal dynamics throughout tumorigenesis. Using the same IHC lesion assessment and quantification methods described for SYP quantification (Figure 3), we stained pancreata from 6 and 12-month-old *KC* mice and *KPC* mice with and without PDAC for each EEC subtype specific hormone. As anticipated, the proportion of each hormone-expressing population per lesion grade followed similar dynamics observed for SYP, with EEC subtype abundance decreasing with increasing lesion grade (Supplementary File S1). For SST expression, 442 and 344 lesions from 6 and 12-month-old *KC* pancreata (*n* = 8 and *n* = 6, respectively) were combined and analyzed as well as 661 lesions from *KPC* (*n* = 19) mice. The percentage of SST+ lesions in *KC* pancreata was significantly higher in metaplastic lesions as compared to PanIN1a (5.49%, *n* = 155 vs 3.91%, *n* = 607; *p* < 0.001) and PanIN1b (2.21%, *n* = 20; *p* < 0.05) (Figure 4A and Supplementary File S1). Only 1 PanIN2 and 2 PanIN3 lesions were captured in the *KC* data set, all of which were negative for SST. For *KPC* (n = 19), the percentage of SST+ cells in metaplastic lesions (3.64%, *n* = 329) was significantly higher than PanIN1a (2.35%, *n* = 220; *p* < 0.01), PanIN1b (0.89%, *n* = 28; *p* < 0.01), PanIN2 (0.11%, *n* = 17; *p* < 0.01), and PanIN3 (0.18%, *n* = 67; *p* < 0.001) (Figures 4A,C and Supplementary File S1). Additionally, the percentage of SST+ cells was significantly higher in PanIN1a lesions versus PanIN3 (*p* < 0.01) further supporting the observed downward trend. PDAC-associated SST+ cells were identified in only two PDAC regions of interest (ROIs, out of 67) from only two *KPC* mice (Figure 4D). These analyses demonstrate that SST+ cells are most abundant in the metaplastic lesions of *KC* and *KPC* pancreata, and that incidence decreases throughout pancreatic tumorigenesis.

In our analyses we also found that the proportion of PP+ cells follow a similar trajectory to SST+ cells throughout pancreatic tumorigenesis where abundance decreases as lesion grade progresses. Lesions totaling 538 and 472 from 6 and 12-month-old *KC* mice (*n* = 9 and *n* = 8, respectively) were assessed. The highest proportion of PP+ cells was observed in the metaplastic lesions (6.07%, *n* = 220), which was significantly higher than PanIN1a (4.01%, *n* = 717; *p* < 0.001) and PanIN1b (2.78%, *n* = 33; *p* < 0.005) (Figure 4B and Supplementary File S1). No PanIN2 or PanIN3 lesions were identified in the *KC* data set. In the *KPC* pancreata (*n* = 19), the proportion of PP+ cells in metaplastic lesions (5.57%, *n* = 336) were significantly higher than PanIN1a (3.85%, *n* = 236; *p* < 0.005), PanIN2 (0.67%, *n* = 37; *p* < 0.001), and PanIN3 (0.59%, *n* = 84; *p* = 0.001) (Figures 4B,C and Supplementary File S1). Additionally, the proportion of PP+ cells was significantly higher in PanIN1a versus PanIN2 (*p* < 0.01) and PanIN3 (*p* < 0.001) lesions further supporting the observed downward trend of PP+ cells as lesion grade progresses. Lastly, four PDAC ROIs (out of 51) from four different *KPC* mice contained PP+ cells (Figure 4D).

**FIGURE 5:**
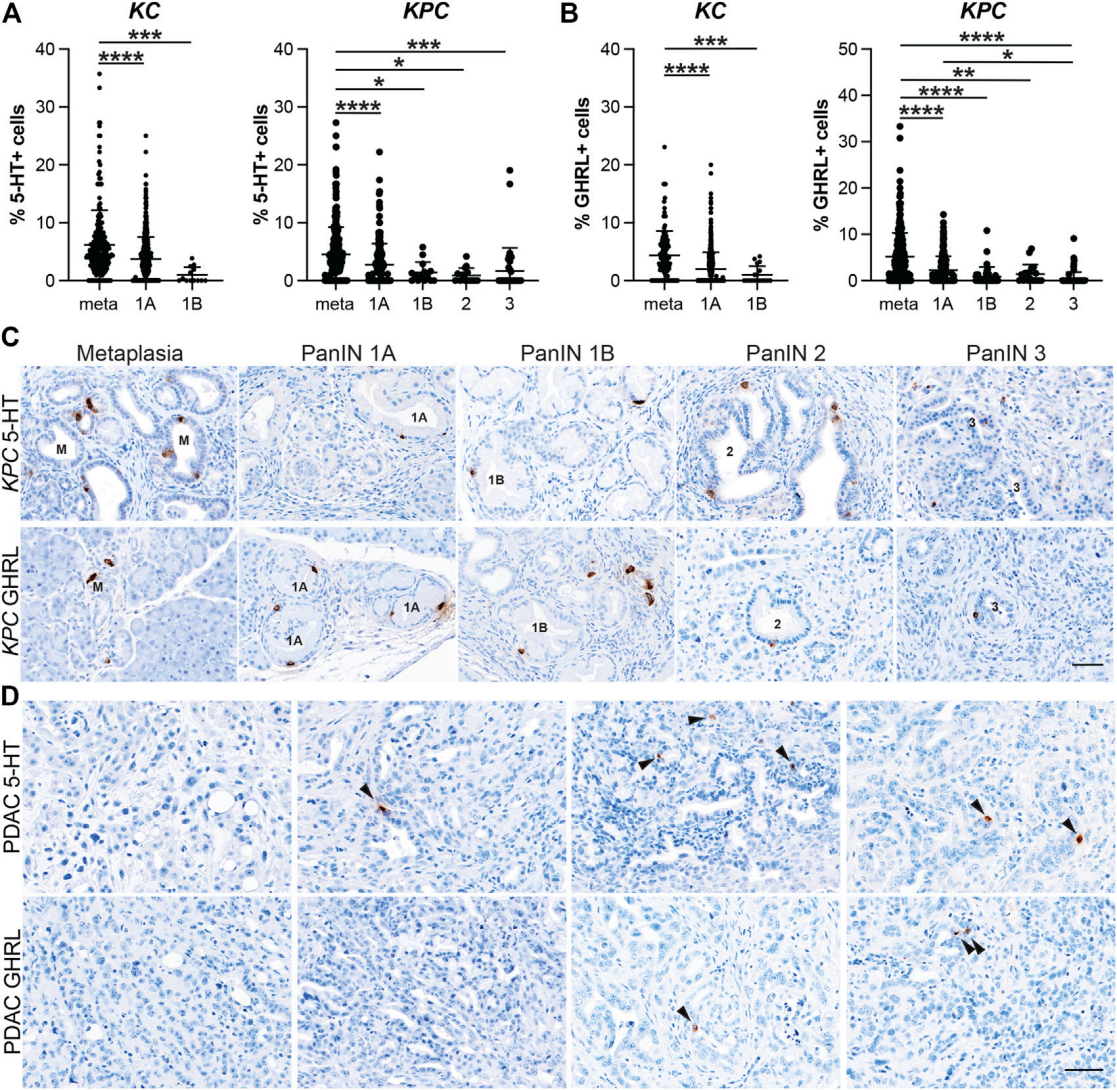
Enterochromaffin and epsilon cell abundance throughout pancreatic tumorigenesis. Quantification of **(A)** enterochromaffin cells (5-HT+) or **(B)** epsilon cells (GHRL+) in lesions from *KC* or *KPC* mice. *p < 0.05; **p < 0.01, ***p < 0.005; ****p < 0.001. **(C)** Representative 5-HT or GHRL IHC of lesions of increasing grade in *KPC* mice. **(D)** IHC for 5-HT or GHRL in adenocarcinoma in *KPC* mice. Arrows, positive cells. Scale bar, 50 μm.

5-HT+ cells exhibited similar dynamics to SST+ and PP+ cells throughout tumorigenesis. Lesions from 6 and 12-month-old *KC* mice (*n* = 8 and *n* = 7, respectively) totaled 457 and 351. The percentage of 5-HT+ cells per lesion was significantly higher in metaplastic lesions (6.18%, *n* = 233) compared to PanIN1a (3.73%, *n* = 562; *p* < 0.001) and PanIN1b (0.98%, *n* = 13; *p* < 0.005) (Figure 5A and Supplementary File S1). No PanIN2 or -3 lesions were identified in this *KC* data set. 572 total lesions were analyzed from *KPC* pancreata with or without PDAC (*n* = 19) and displayed a similar trend to the *KC* data. Metaplastic *KPC* lesions contained a significantly higher percentage of 5-HT+ cells per lesion (4.54%, *n* = 290) compared to PanIN1a (2.75%, *n* = 213; *p* < 0.001), PanIN1b (1.42%, *n* = 14; *p* < 0.05), PanIN2 (0.89%, *n* = 14; *p* < 0.05), and PanIN3 (1.65%, *n* = 41; *p* < 0.005) (Figures 5A,C and Supplementary File S1). Interestingly, 34/88 5-HT+ PDAC ROIs were identified in seven of nine tumor bearing *KPC* mice compared to SST (*n* = 2), PP (*n* = 4), and GHRL (*n* = 5) (Figure 5D). This observation suggests differing roles for each hormone throughout different stages of tumorigenesis.

Lastly, the dynamics of GHRL+ cells followed a similar course as the previously described hormones. 374 and 296 lesions from 6 and 12-month-old *KC* mice (*n* = 7 and *n* = 5, respectively) were analyzed, and metaplastic ducts contained a significantly higher proportion of GHRL+ positive cells (4.34%, *n* = 122) as compared to PanIN1a (2.00%, *n* = 517; *p* < 0.001) and PanIN1b (1.00%, *n* = 18; *p* < 0.005) (Figure 5B and Supplementary File S1). There were no PanIN2 or -3 lesions identified in this *KC* dataset. The *KPC* (*n* = 19) dataset exhibited the same trend where the percentage of GHRL+ cells significantly decrease as lesion grade increases. Metaplastic *KPC* lesions contained a significantly higher percentage of GHRL+ cells (5.19%, *n* = 337) as compared to PanIN1a (2.25%, *n* = 168; *p* <0.001), PanIN1b (0.87%, *n* = 38; *p* < 0.001), PanIN2 (1.45%, *n* = 19; *p* < 0.01), and PanIN3 (0.41%, *n* = 70; *p* < 0.001) (Figures 5B,C and Supplementary File S1). Additionally, the percentage of GHRL+ cells was significantly higher in PanIN1a versus PanIN3 (*p* < 0.05) further supporting the observed downward trend. GHRL+ cells were identified in 5 PDAC ROIs (*n* = 64) from two different *KPC* mice (Figure 5D). Altogether, the expression of each EEC subtype hormone is most abundant in metaplasia and significantly decreases with tumor progression. EEC subtype abundance in 6- vs 12-month-old *KC* mice and between *KC* and *KPC* mice varies by subtype (Supplementary Figure S3).

### PanIN Are Constituted by Distinct Enteroendocrine Cell Subtypes

**FIGURE 6:**
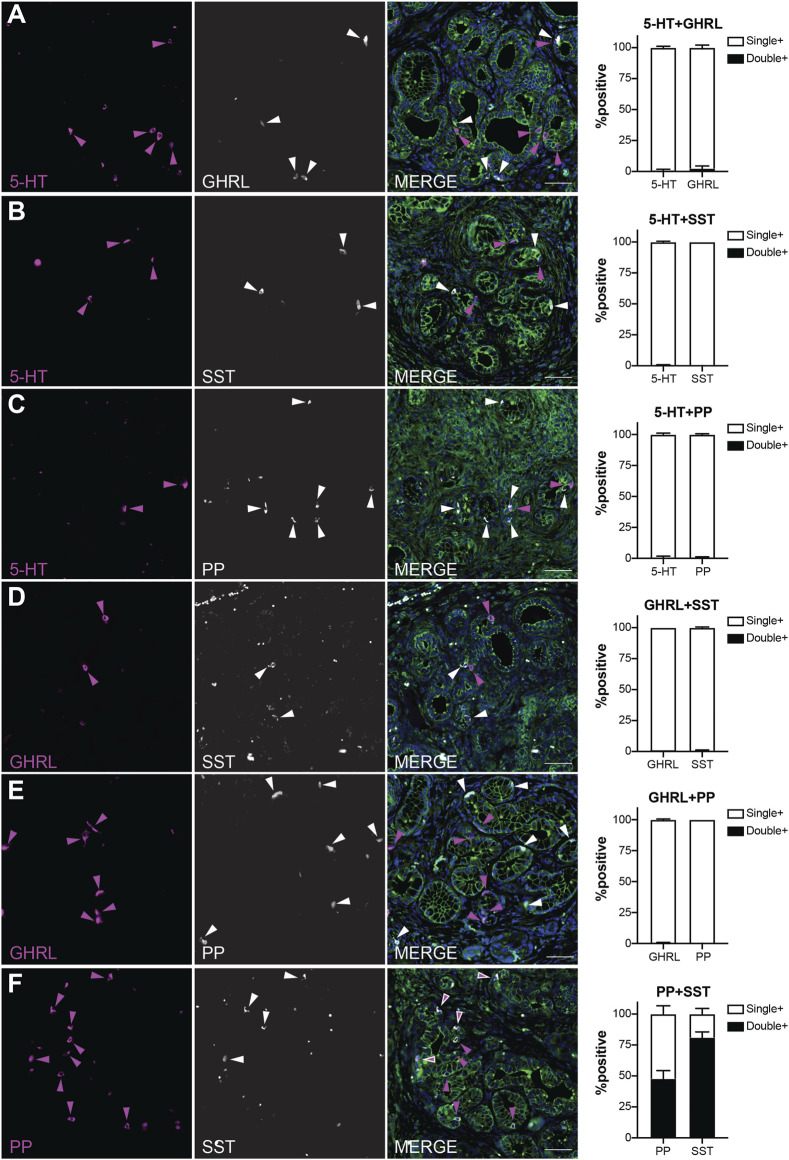
EEC subtypes identified by distinct and overlapping hormone signatures. Co-immunofluorescence and quantification of **(A)** 5-HT and ghrelin (0.67 and 2.33% co-expression, respectively), **(B)** 5-HT and SST (0.33 and 0% co-expression, respectively), **(C)** 5-HT and PP (0.67 and 0.67% co-expression, respectively), **(D)** ghrelin and SST (1 and 0.67% co-expression, respectively), **(E)** PP and ghrelin (0 and 0.33% co-expression, respectively), and **(F)** PP and SST (47.7 and 81% co-expression, respectively). Scale bar, 50 μm.

Our recently published scRNA-seq study of injury-induced pancreatic ADM identified multiple EEC subtypes based on individual hormone expression (Ma et al., 2021). Based on these results, we hypothesized that hormone expression in pancreatic tumorigenesis is largely EEC subtype restricted. To test this hypothesis, we performed co-IF on 6-month-old *KC* pancreata (*n* = 3 mice) for the previously described EEC hormones to determine if the EECs arising in tumorigenesis represent distinct lineages. Pancreata in this analysis harbored primarily ADM and PanIN1a lesions and were co-stained with 2-hormone combinations and γ-actin which served as a membranous marker. 100 non-islet associated, hormone positive cells were identified for each staining panel per mouse (300 total cells), then dual-hormone expression was identified and quantified (*see* methods). Most hormone expression in 6-month-old *KC* pancreata is subtype-restricted, with a co-positivity rate of 2.3% or less (Figure 6). Only 0.67% of 5-HT+ cells were co-positive for GHRL, and 2.33% GHRL+ were co-positive for 5-HT (Figure 6A). Similar results were observed between 5-HT and SST, with 0.33% of 5-HT+ cells co-positive for SST, and 0% of SST+ cells co-positive for 5-HT (Figure 6B). 0.67% of 5-HT+ and PP+ cells were co-positive (Figure 6C). 1% of GHRL+ cells were co-positive for SST, and 0.67% of SST+ cells were co-positive for GHRL (Figure 6D). 0% of PP+ cells were co-positive for GHRL, and 0.33% of GHRL+ cells were co-positive for PP (Figure 6E). This confirms our hypothesis that hormone expression is largely EEC subtype restricted.

In contrast, PP and SST showed a high degree of co-expression with 81% of SST+ cells co-positive for PP, and 47.7% of PP+ cells co-positive for SST (Figure 6F). This high degree of overlap could be due to the common lineage trajectory identified for these two EEC subtypes in our previous work or could be the formation of bihormonal ADM-derived gamma cells similar to those in islets recently reported by Perez-Frances et al. (Ma et al., 2021; Perez-Frances et al., 2021). Additionally, our scRNA-seq data predicts that a subset of enterochromaffin cells co-express gastrin, a hormone secreted by G cells located in the antrum of the stomach and duodenum (Yacoub et al., 1996; Ma et al., 2021). Co-IF analysis of 5-HT and gastrin identified 15% of 236 gastrin+ cells positive for 5-HT, and 9.33% of 300 5-HT+ cells as co-expressing gastrin (Supplementary Figure S4). Together, these observations support the hypothesis that EEC hormone expression is primarily restricted to its respective subtype, but also identifies dual PP-SST and 5-HT-gastrin expressing EECs in pancreatic tumorigenesis.

### POU2F3 Is Not Required for EEC Formation

**FIGURE 7:**
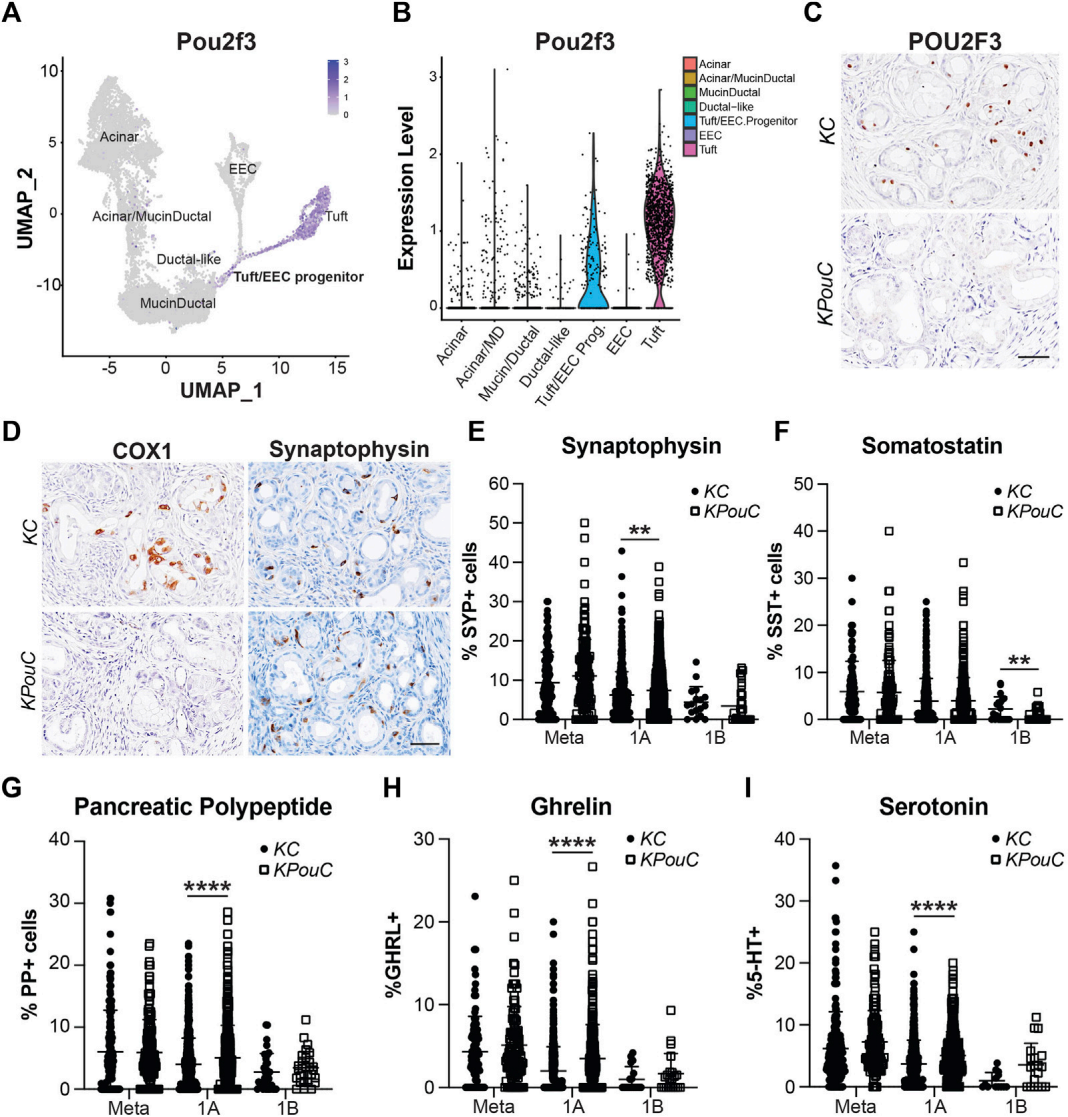
POU2F3 is not required for EEC formation but affects abundance. **(A)** UMAP and **(B)** Violin plot of *Pou2f3* expression in an injury-induced pancreatic metaplasia dataset demonstrating expression in tuft cells as well as the predicted tuft/EEC common progenitor population. Modified from Ma et al. **(C)** IHC for POU2F3 or **(D)** COX1 or SYP in 6-month-old *KC* or *KPouC* pancreata. Scale bar, 50 μm. **(E)** Quantification of pan-EEC marker synaptophysin (SYP) and EEC subtype markers **(F)** SST, delta cells, **(G)** PP, gamma cells, **(H)** GHRL, epsilon cells, and **(I)** 5-HT, enterochromaffin cells, in the pancreata of 6- and 12-month-old *KC* or *KPouC* mice. **p < 0.01; ****p < 0.001.

Transcription factor POU2F3 is the master regulator of tuft cell formation in normal organs and in pancreatic tumorigenesis (Gerbe et al., 2016; DelGiorno et al., 2020b). Our scRNA-seq study of pancreatic ADM identified *Pou2f3* expression in a progenitor cell population predicted to seed both tuft cells and EECs (Figures 7A,B) (Ma et al., 2021). Comparison of our dataset with a scRNA-seq study of *Kras* ^*G12D*^-induced ADM suggests this population is present in pancreatic tumorigenesis as well (Supplementary Figure S5A) (Ma et al., 2021; Schlesinger et al., 2020). To determine if POU2F3 is required for EEC formation in pancreatic tumorigenesis, we examined EEC abundance in the pancreata of *Kras* ^*G12D*^ *;Pou2f3* ^*fl/fl*^ *;Ptf1a* ^*Cre/+*^ (*KPouC*, tuft cell knockout) mice aged to 6 or 12-months (DelGiorno et al., 2020b). IHC staining for POU2F3 demonstrates a lack of expression in *KPouC* pancreata (Figure 7C). Additionally, COX1, a marker of mature tuft cells, is absent from the epithelium of *KPouC* pancreata, consistent with a lack of tuft cell formation (Figure 7D) (Grunddal et al., 2021; Ma et al., 2021; Manco et al., 2021). Interestingly, SYP+ cells were identified in both *KC* and *KPouC* pancreata indicating that EEC formation is not prevented by the loss of POU2F3 (Figure 7D). Further, all EEC subtype-specific hormones are also present in *KPouC* pancreata and display the same general downward trend in abundance as lesion grade increases (Supplementary Figure S5). These data demonstrate that ablating *Pou2f3* in the context of an activating *Kras* ^*G12D*^ mutation does not prevent EEC formation.

### Loss of POU2F3 Impacts EEC Subtype Abundance

To determine if loss of POU2F3 affects the dynamics of EEC subtype formation and abundance, we executed the same IHC quantification strategy used for *KC* and *KPC* mice (Figures 3–5). SYP IHC staining of combined 6 and 12-month *KPouC* pancreata (*n* = 13) exhibited the same trend as in the *KC* and *KPC* models, with metaplastic lesions containing a significantly higher percentage of SYP+ cells (11.10%, *n* = 139) as compared to PanIN1a (7.44%, *n* = 562; *p* < 0.001), PanIN1b (3.47%, *n* = 28; *p* < 0.001), PanIN2 (0%, *n* = 15; *p* < 0.001), and PanIN3 (0%, *n* = 12; *p* < 0.001) (Supplementary Figure S5B and Supplementary File S1). As compared to *KC*, *KPouC* PanIN1a lesions contain a significantly higher percentage of SYP+ cells (7.44%, *n* = 562 vs 6.21%, *n* = 505; *p* < 0.01), while SYP+ cells are similarly abundant in metaplasia and PanIN1b (Figure 7E). Differences were also identified in EEC subtype abundance between the tuft cell+ (*KC*) and tuft cell knockout (*KPouC*) mice. Delta/SST+ cell abundance is significantly lower in *KPouC* vs *KC* PanIN1b lesions (0.68%, *n* = 37 vs 2.12%, *n* = 20; *p* < 0.01) (Figure 7F and Supplementary File S1). In terms of PanIN1a, *KPouC* mice have a significantly higher abundance of PP (5.07%, *n* = 617 vs 4.01%, *n* = 717; *p* < 0.001), GHRL (3.48%, *n* = 460 vs 2.00%, *n* = 517; *p* < 0.001), and 5-HT (5.09%, *n* = 420 vs 3.73%, *n* = 562; *p* < 0.001) cells (Figures 7G–I and Supplementary File S1). These differences may be attributed to the faster rate of tumorigenesis in *KPouC* versus *KC* mice which is supported by the identification of more PanIN2 and PanIN3 lesions in *KPouC* pancreata (Supplementary File S1) (DelGiorno et al., 2020b). Alternatively, POU2F3 or tuft cells may play a role, directly or indirectly, in specifying EEC subtype formation.

### Enteroendocrine Cells as a Cellular Compartment of the Normal Human Pancreas

**FIGURE 8:**
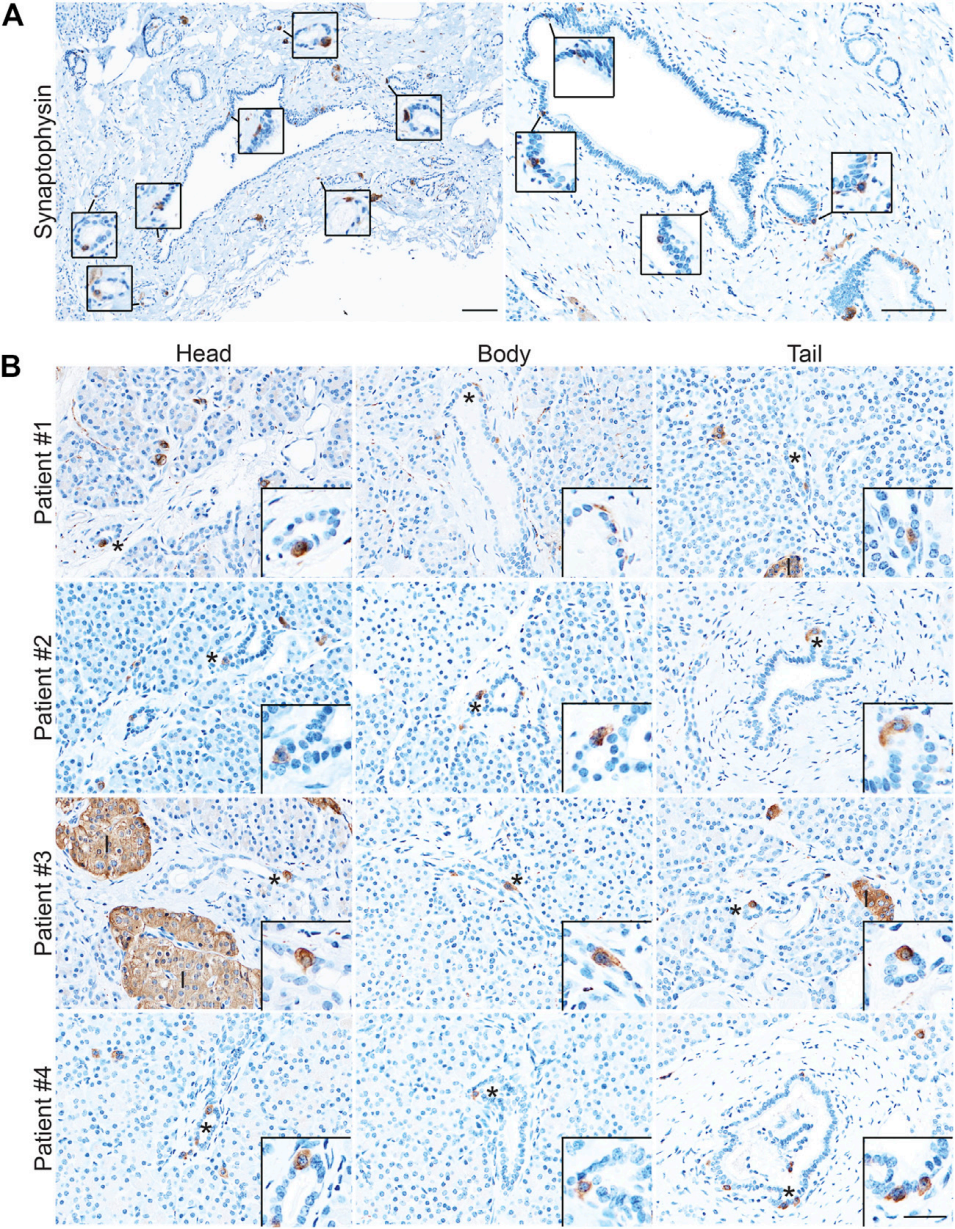
Enteroendocrine cells as a cellular compartment of the normal human pancreas. Synaptophysin IHC of **(A)** large, pathologically normal ducts in human pancreata. Scale bar, 100 μm. **(B)** Synaptophysin IHC of pancreata from four human donors determined to have normal islet function. Images are representative of the head, body, and tail of the same pancreata. Scale bars, 50 μm for main images, 25 μm for inserts.

**FIGURE 9:**
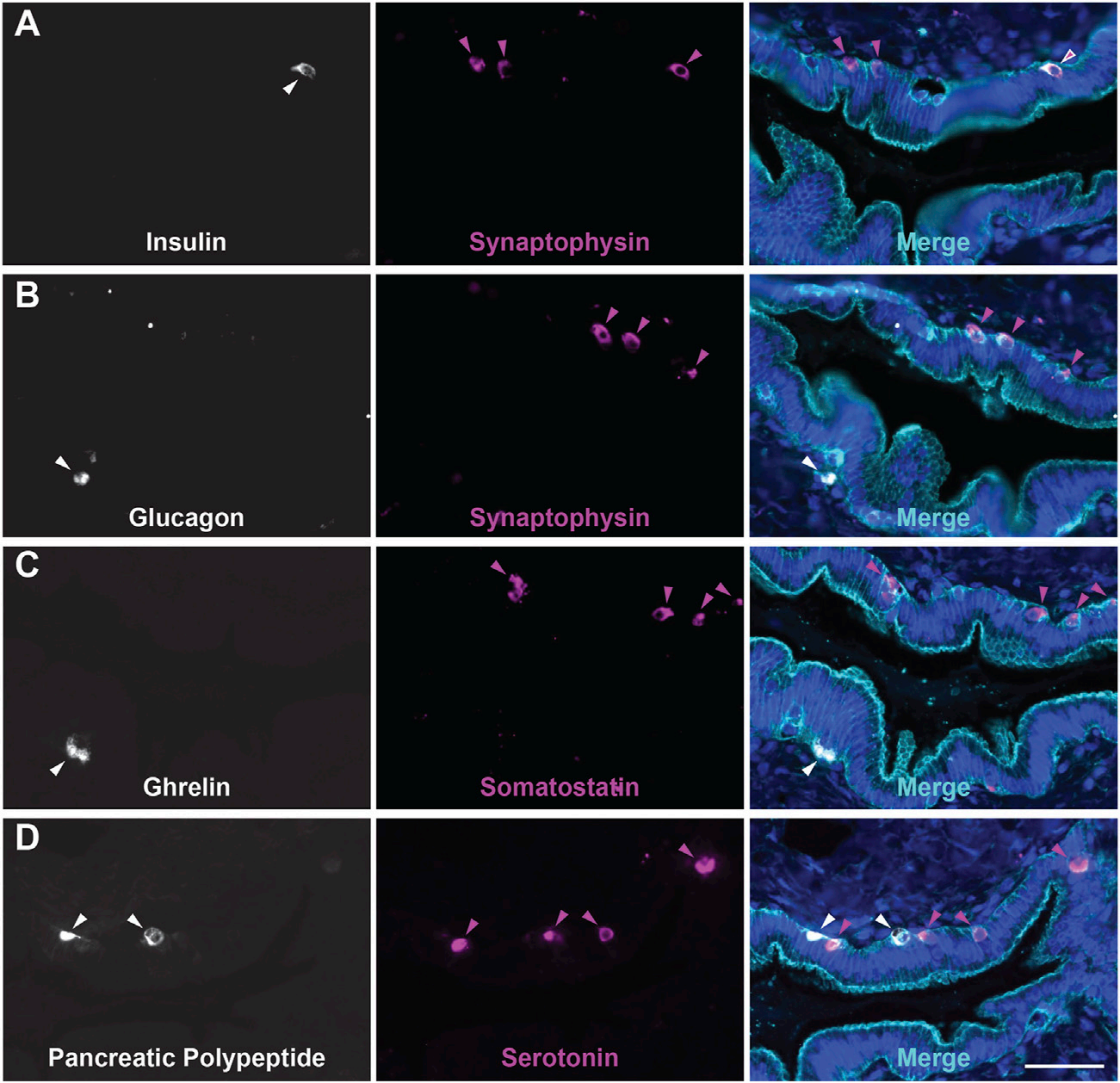
Endocrine/Enteroendocrine cell subtypes in the ducts of the human pancreas. Co-immunofluorescence for hormones **(A)** INS (white) and SYP (magenta), **(B)** GCG (white) and SYP (magenta), **(C)** GHRL (white) and SST (magenta), or **(D)** PP (white) and 5-HT (magenta), and γ Actin (cyan) and DAPI (blue). Scale bar, 50 μm.

While the presence of solitary hormone secreting cells has been described in the normal pancreas, injured pancreas, Type 1 diabetes, and cystic fibrosis, the characterization of EECs in the normal pancreas is incomplete (Chen et al., 1988; Brissova et al., 2018; Hart et al., 2018; Ma et al., 2021). To determine if normal human ducts are populated by EECs, we assessed three distinct regions (head, body, tail) of normal human donor pancreata (*n* = 4) for the presence of SYP and EEC subtype markers. As shown in Figure 8A, we identified SYP+ cells within the epithelial layer of pathologically normal large ducts, as well as in surrounding basal glands. Additionally, SYP+ cells were identified in smaller, intra- and interlobular ducts throughout the head, body, and tail of all pancreata, supporting the observation that EECs are a cellular compartment of the normal human pancreas (Figure 8B). To determine if human pancreata are populated by the EEC subtypes described in this study, we performed co-IF for GHRL, SST, PP, and 5-HT, as well as INS (beta cells) and GCG (alpha cells) (Figure 9). Interestingly, we identified solitary INS+ and GCG+ cells within ducts in the head (INS, ¾ pancreata; GCG _2/4_ pancreata), body (INS, ¼ pancreata; GCG ¼ pancreata), and tail (INS, ¼ pancreata; GCG _2/4_ pancreata) (Figures 9A,B). Whether these cells are associated with islets in another plane could not be determined, however, these solitary cells are part of the single layer epithelium of these ducts. Next, we assayed pancreata for EEC subtype hormones and could identify expression in the head (SST, ¾; PP, ¼; 5-HT, ¼), body (GHRL, ¼; SST, _2/4_; PP, ¾; 5-HT, ¼), and tail (GHRL, ¾; SST, ¼; PP, _2/4_; 5-HT, _2/4_) (Figures 9C,D). Altogether all EEC subtype hormones were identified in the ducts of normal human pancreata, supporting the identification of EECs as a cellular compartment of the normal human pancreas. Whether or not these cells have a distinct origin from islets remains to be determined.

### Dynamics of EEC Subtype Abundance in Human Pancreatic Tumorigenesis

Expression of endocrine hormones has been reported in human PanIN and PDAC, however the formation and dynamics of EEC subtypes throughout tumorigenesis has not been assessed (Farrell et al., 2017; Sinha et al., 2017; Chen et al., 1988; Sakaki et al., 2002). Here, we performed co-IF for SST/γ-actin/GHRL (*n* = 21 slides) and 5-HT/γ-actin/PP (*n* = 20 slides) on human PanIN and PDAC samples (*n* = 11 patients), as well as adjacent normal, and identified ductal lesions containing hormone+ cells. These lesions were then graded by a pathologist. Ducts were classified as normal or reactive (Supplementary Figure S6A) and lesions were classified by grade (ADM, PanIN1a, PanIN1b, PanIN2, PanIN3, invasive adenocarcinoma). Additionally, ADM-like ductal structures residing within a lobule harboring a higher graded lesion were termed a “basal gland” of that lesion. These glands are similar to the glands associated with the pancreatic and common bile ducts, and therefore may be considered an extension of the larger lesion (*see* Supplementary File S2 for classification definitions) (Yamaguchi et al., 2015). As we cannot confirm the origin of basal glands in our 2D analysis, these structures were analyzed separately from their associated ductal lesion (Supplementary Figure S6B and Supplementary File S2).

**FIGURE 10:**
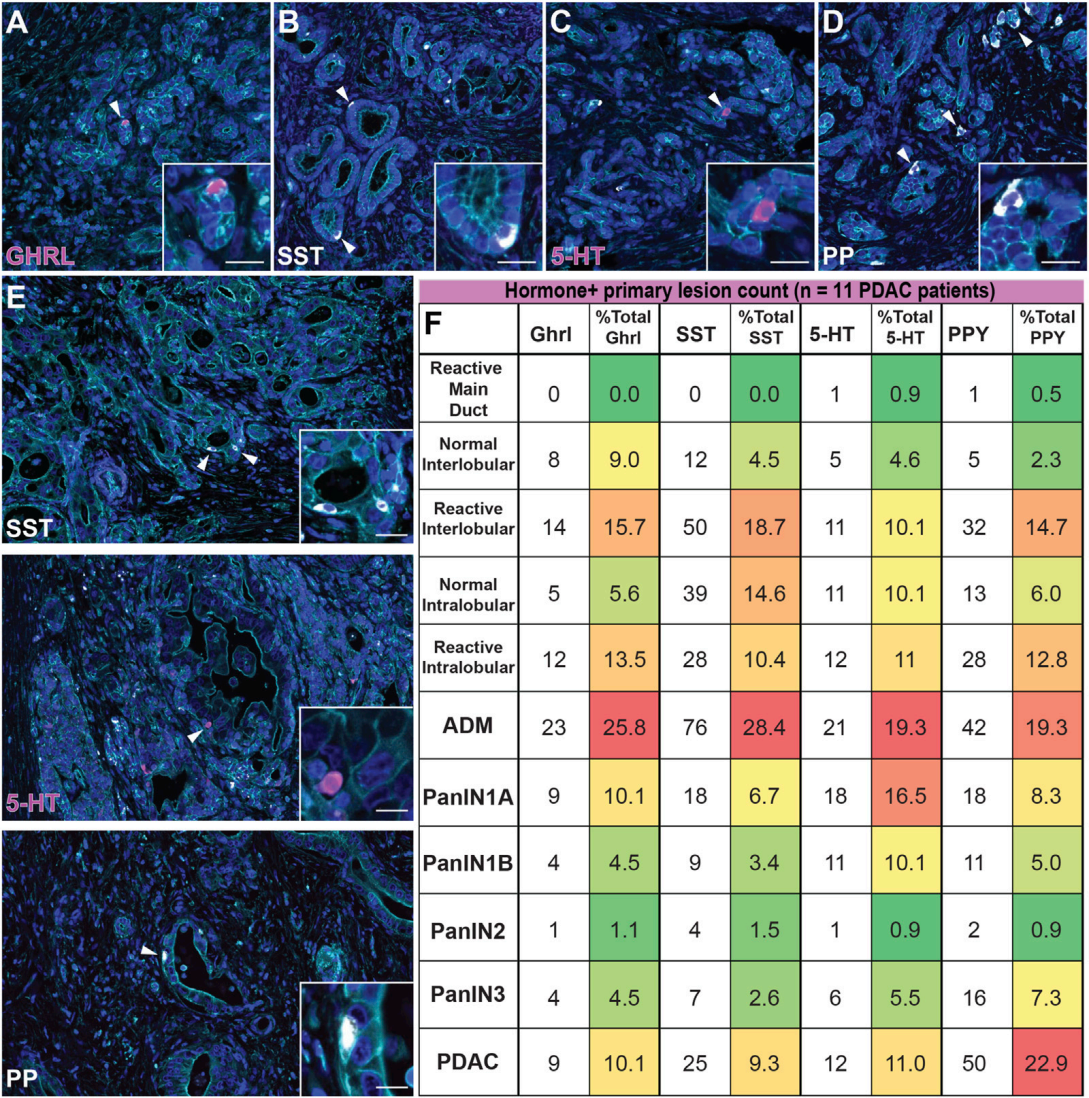
Enteroendocrine cell subtype dynamics throughout human pancreatic tumorigenesis. Co-immunofluorescence for **(A)** GHRL (magenta), **(B)** SST (white), **(C)** 5-HT (magenta), or **(D)** PP (white) and γactin (cyan), and DAPI (blue) in human ADM lesions. **(E)** Co-immunofluorescence for SST (white), 5-HT (magenta), or PP (white) and γactin (cyan), and DAPI (blue) in human adenocarcinoma. Scale bar, 50 μm. **(F)** Table depicting the number of human lesions harboring at least one hormone+ cell for each EEC subtype and organized by lesion grade. The number of EEC-containing lesions were totaled per hormone, and the relative distribution (%) of EEC-containing graded lesions are displayed (11 patients); red = highest % of total; green = lowest % of total.

The percentage of each hormone+ lesion class (e.g. normal, PanIN, etc.) relative to the total number of positive lesions for that hormone were calculated and analyzed. Consistent with our mouse data set, we observed similar dynamics throughout tumorigenesis for each hormone analyzed. ADM lesions represented the highest percentage of GHRL+ (25.8%), SST+ (28.4%), 5-HT+ (19.3%), and PP+ (19.3%) lesions relative to the average of combined normal inter- and intralobular ducts (7.3, 9.6, 7.4, and 4.2%, respectively) and high grade (PanIN3, 4.5, 2.6, 5.5, and 7.3%, respectively) for each hormone (Figures 10A–D,F). In general, the relative proportion of hormone+ lesions decreased as lesion grade increased. Overall, *KC* and *KPC* mouse data recapitulate EEC dynamics observed in human tumorigenesis.

Upon further analysis of each individual hormone, we observed notable differences in EEC dynamics within the human data set as compared to mouse models. Of the non-lesion ducts (normal and reactive main, inter-, and intralobular), reactive interlobular ducts harbored the highest percentage GHRL+ (15.7%), SST+ (18.7%), and PPY+ (14.7%) lesions (Supplementary Figure S6A). Additionally, we observed more hormone+ reactive inter- and intralobular ducts as compared to their normal counterparts (Figure 10F). Interestingly, we observed that the percentage of 5-HT+ lesions is largely represented by ADM and PanIN1a, with a notable, but less pronounced increase in invasive adenocarcinoma (Figures 10E,F). Lastly, the abundance of PP+ lesions most notably fluctuates over the course of tumorigenesis, as compared to the other hormone+ lesions. While the proportion of hormone+ invasive adenocarcinoma lesions is relatively high for each individual hormone (10.1% of total GHRL+, 9.3% of total SST+, and 11.0% of total 5-HT+ lesions) we observed a more exaggerated second peak of PP-harboring invasive adenocarcinoma lesions (22.9% of total PP+ lesions) (Figures 10E,F). These observations demonstrate the dynamic nature of EECs throughout human tumorigenesis.

## Discussion

Early events in pancreatic tumorigenesis are poorly understood. Here, we used histological methods to describe and quantify the dynamics of EEC subtype (gamma, delta, epsilon, and enterochromaffin cell) formation and abundance throughout tumorigenesis in murine models and human disease (Ma et al., 2021). We found, in both murine and human pancreata, that EEC subtypes are most abundant in ADM and PanIN1a lesions and that these cells decrease in frequency with disease progression. In human disease we observed a moderate resurgence of EEC subtype abundance in invasive adenocarcinoma relative to pre-invasive lesions suggesting that these cells may have different functional roles in different stages of tumorigenesis. Additionally, we quantified previously undescribed bihormonal EECs (gastrin and 5-HT, SST and PP) in 6-month-old *KC* pancreata, the latter of which has been described only in islets (Perez-Frances et al., 2021). This bihormonal expression reflects either the maturation state of this population, or a functional subclass of these EECs. We also show that tuft cell master regulator transcription factor POU2F3 is not required for EEC formation in *KC* mice but does affect the abundance of EEC subtypes in early lesions. This could be due to the direct actions(s) of tuft cell secretory products on EEC formation or could be an indirect response to the stromal changes that result from tuft cell loss (DelGiorno et al., 2020b). Collectively, these data demonstrate that EEC subtype formation within *Kras* ^*G12D*^-induced epithelial lesions is an early event in pancreatic tumorigenesis and that these cells likely play different functional roles throughout tumor progression.

Interestingly, we also observed differences in the abundance of each EEC population throughout tumorigenesis and between mouse models and human disease. In mice, 34 of 88 PDAC ROIs contain 5-HT+ cells, the highest of all subtypes analyzed. In human disease, ADM, PanIN1a, and PanIN1b contain the highest proportion of 5-HT+ lesions while other hormone+ lesions decrease in PanIN1b. Additionally, the proportion of 5-HT+ lesions moderately increase again in invasive adenocarcinoma. Previous studies have shown that 5-HT supports pancreatic tumor growth and modulates inflammation (Jiang et al., 2017; Schneider et al., 2021; Wang et al., 2021). In the context of our findings, we predict that the increase in 5-HT+ enterochromaffin cells in ADM and early PanIN is a reaction to metaplasia and initially inhibits tumorigenesis, but in later stages supports the formation of more aggressive lesions (Soll et al., 2010; Sarrouilhe and Mesnil, 2019). Further studies are required to elucidate the function of 5-HT in tumorigenesis and may also identify therapeutic routes for early intervention.

In our analyses, we observed a relatively high proportion of PP+ and SST+ EECs in normal and reactive pancreatic ducts in the human data set, consistent with prior studies of the normal human pancreatic ductal tree (Li et al., 2016). We also observed an increase in PP+ gamma cells in invasive adenocarcinoma relative to both pre-invasive PP+ lesions and other hormone+ lesions. PP regulates endocrine and exocrine secretion in the normal pancreas and can stimulate certain neuronal pathways (Whitcomb et al., 1990). In pancreatic tumorigenesis, PP may control the function of other secretory cells, such as tuft cells and the EECs described here. However, the function of PP in cancer in unknown and warrants further investigation. In contrast, SST is known to inhibit secretory processes and other cellular processes. SST analogs such as Octreotide and Lanreotide are currently used for the treatment of multiple diseases, including certain cancers (particularly neuroendocrine tumors), which supports a role for SST in downregulating pro-tumorigenic processes in the pancreas (Enzler and Fojo, 2017).

Finally, we report the presence of GHRL+ epsilon cells within the peribiliary glands of the pancreatobiliary duct, a glandular structure reported to serve as a possible progenitor niche, as well as expansion of this population in *Kras* ^*G12D*^-induced tumorigenesis (Yamaguchi et al., 2015). While considered a differentiated cell type in the stomach and intestines, functioning in glucose metabolism (among other roles), GHRL+ cells have also been shown to be a progenitor population in the developing islet (Wren et al., 2001; Tong et al., 2010; Arnes et al., 2012; Poher et al., 2018). Recently, ghrelin was shown to downregulate ductal and fibrotic markers in a genetic model of cholestasis, consistent with an anti-inflammatory role in disease progression (Petrescu et al., 2020). Our data demonstrate that GHRL+ cells are more abundant early in disease progression in both mouse and human, and when coupled with these studies are consistent with a role for ghrelin in mitigating tumorigenesis.

While the number of lesions included in this analysis is extensive, we recognize that 2D histological methods may have captured a single islet cell adjacent to the basement membrane of the ductal epithelium. To limit this possibility, we took care to exclude hormone+ cells not within the epithelial sheet in our analyses. Additionally, without lineage tracing we recognize that we cannot confirm the origin of these EECs (e.g. from acinar cells), however we expect that inclusion into ADM and PanIN would lead to similar functional effects despite cell of origin. Furthermore, despite positive stains for various hormones, evidence of hormone secretion has not yet been demonstrated, and the physiological function of these EEC subtypes remains be investigated.

Altogether, our study identifies and quantifies the dynamics of EEC subtype formation throughout murine and human pancreatic tumorigenesis. In contrast to the endocrine-islet compartment of the pancreas, this enteroendocrine compartment represents an understudied population with a potentially huge impact on pancreas function and disease progression. Further studies are required to determine the individual and combined role(s) of these EEC subtypes and their respective hormones. Manipulation of these pathways alone or in combination with standard chemotherapy may provide more efficacious treatments for PDAC.

## Data Availability

The original contributions presented in the study are included in the article/Supplementary Material, further inquiries can be directed to the corresponding author.
