# Supplementary figures and images for: Enteroendocrine Cell Formation Is an Early Event in Pancreatic Tumorigenesis
Source: Front Physiol. 2022 Apr 27;13:865452. doi: 10.3389/fphys.2022.865452 (PMC9091171; doi:10.3389/fphys.2022.865452)

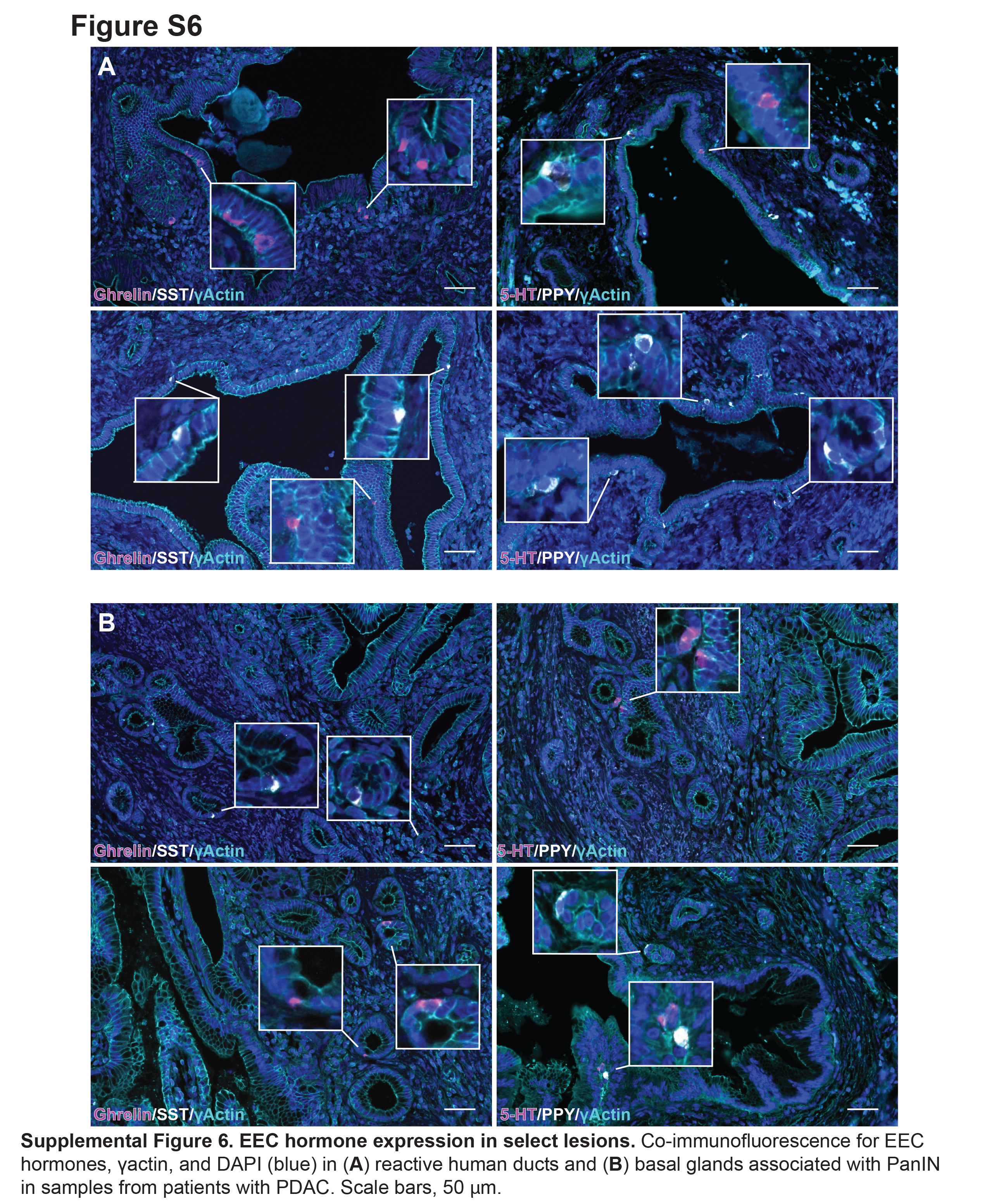

Supplement: Supplementary file 2 [file Image6.TIF]

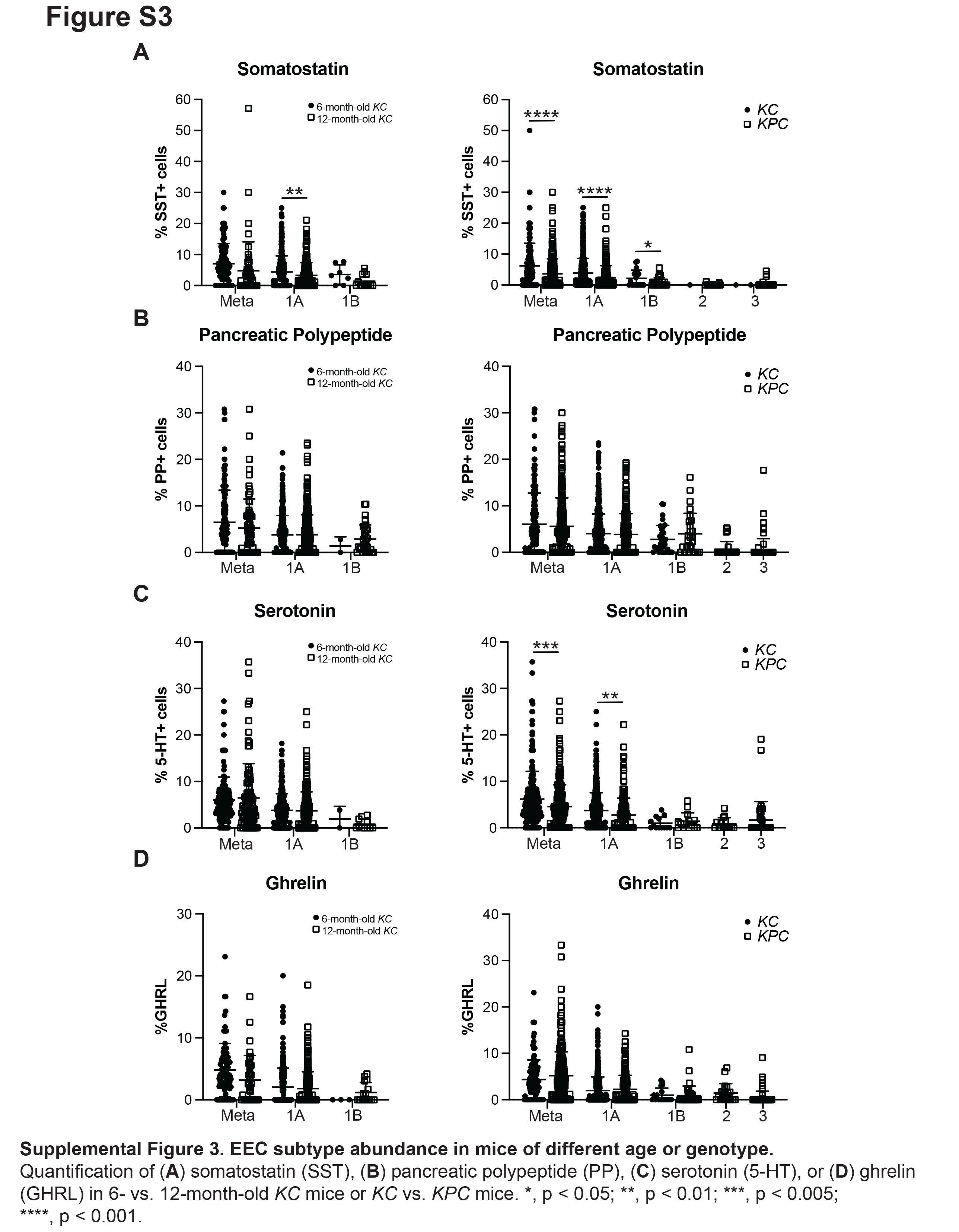

Supplement: Supplementary file 3 [file Image3.TIF]

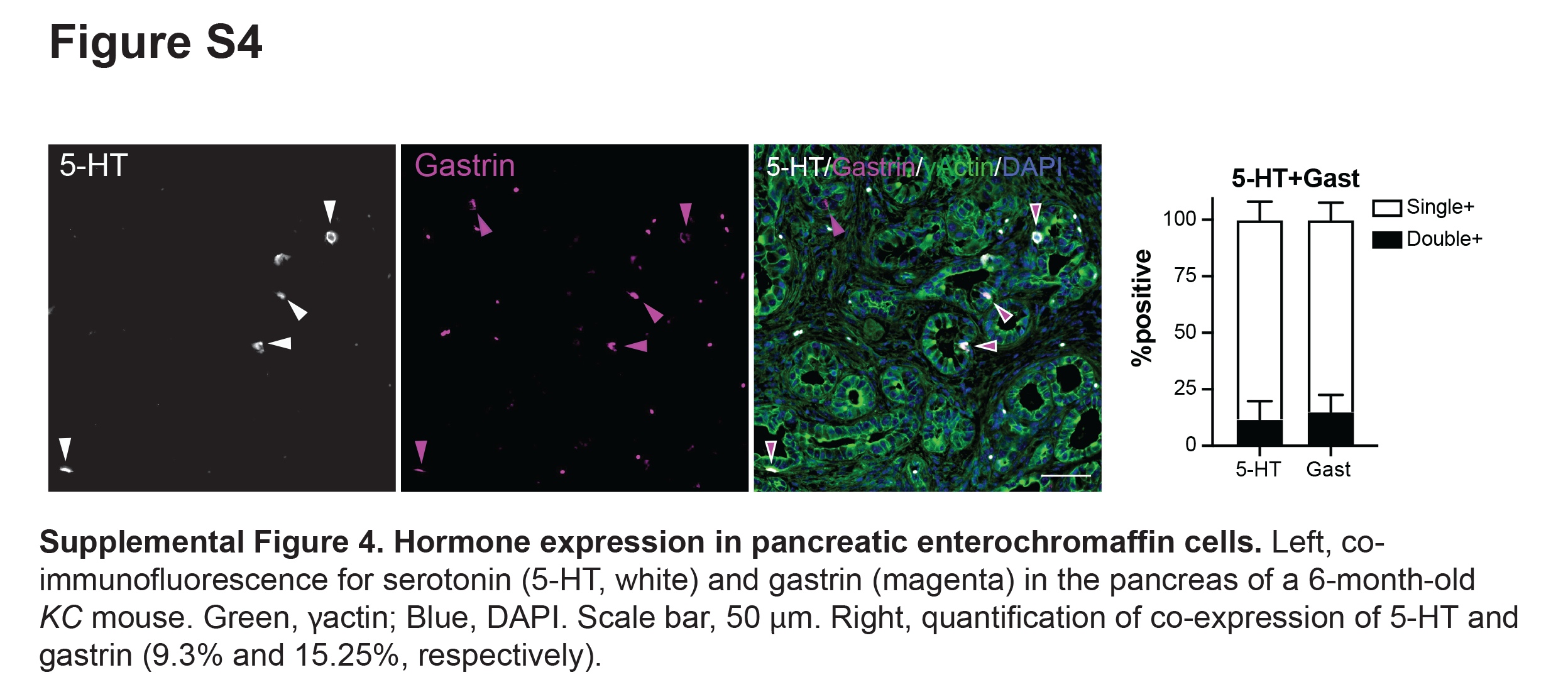

Supplement: Supplementary file 4 [file Image4.TIF]

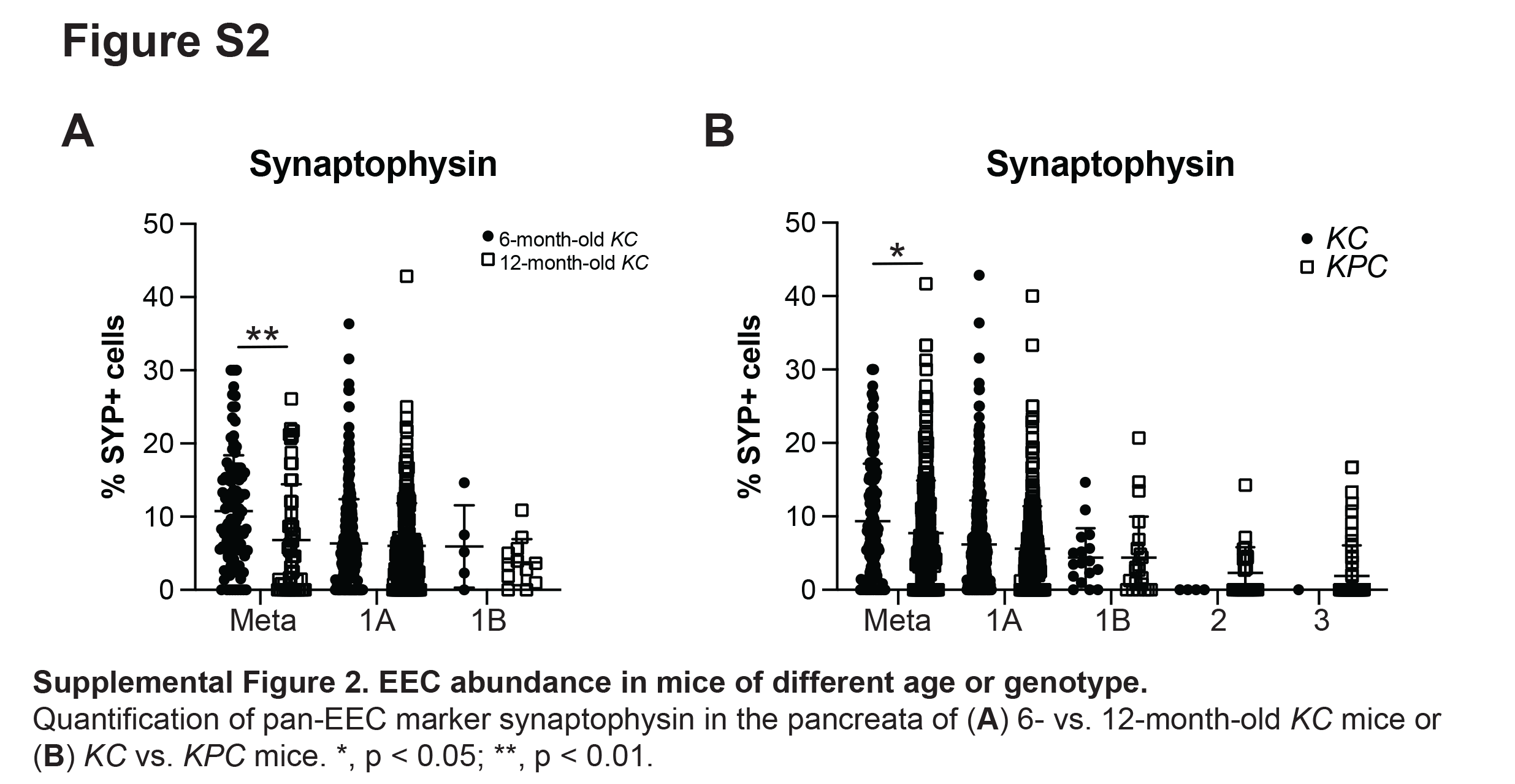

Supplement: Supplementary file 5 [file Image2.TIF]

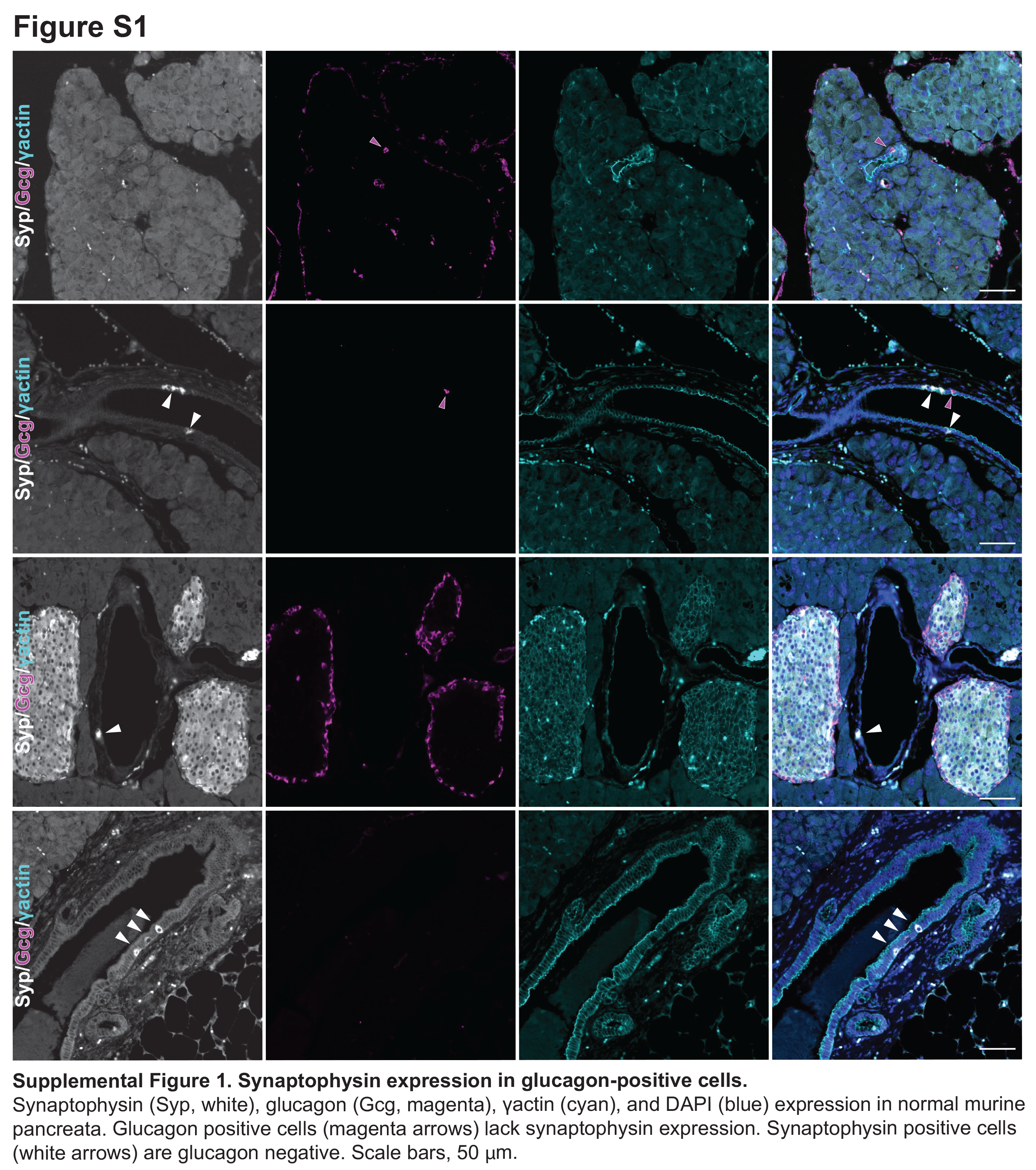

Supplement: Supplementary file 6 [file Image1.TIF]

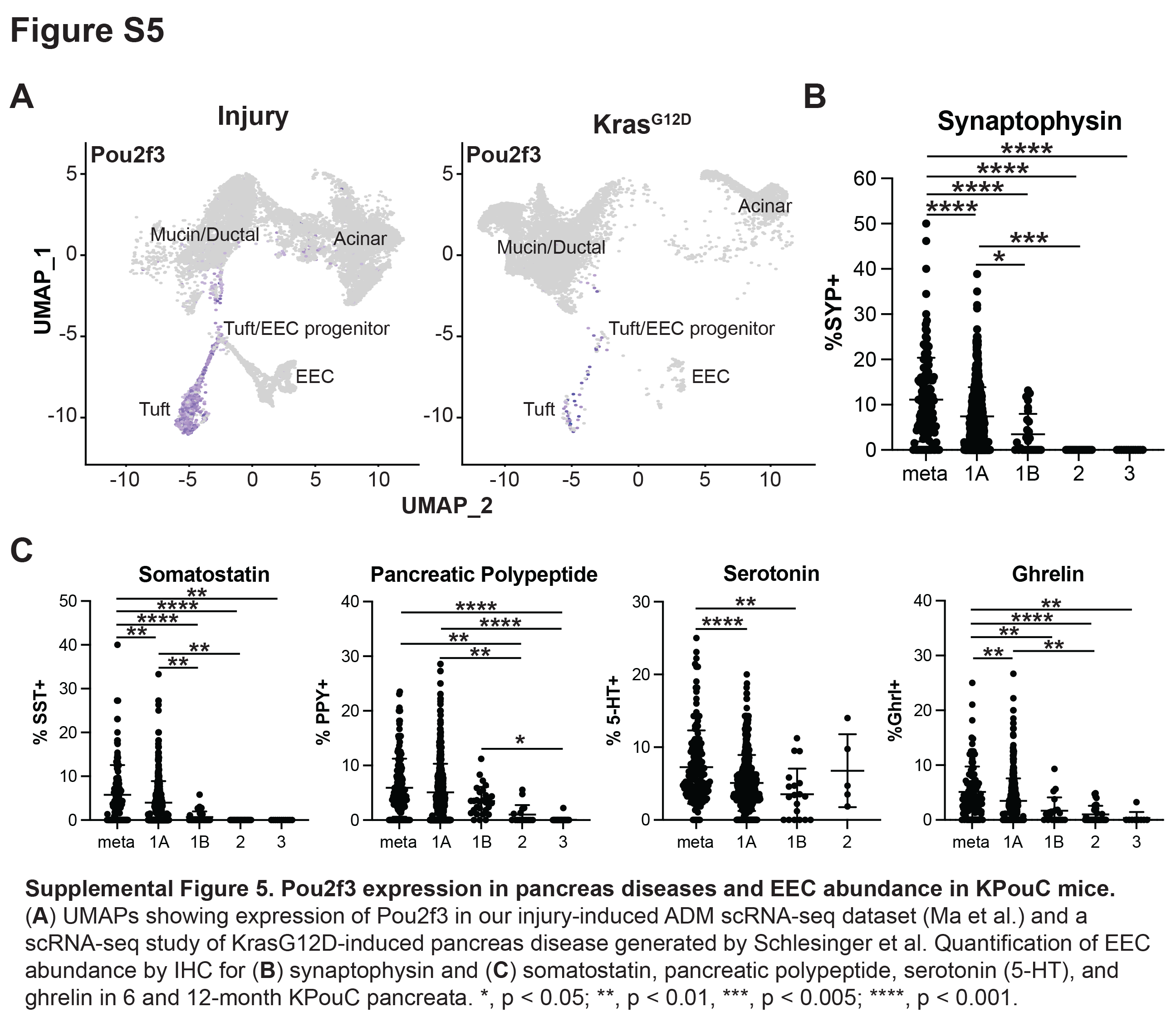

Supplement: Supplementary file 8 [file Image5.TIF]
